# Long-term association of a transcription factor with its chromatin binding site can stabilize gene expression and cell fate commitment

**DOI:** 10.1073/pnas.2000467117

**Published:** 2020-06-12

**Authors:** J. B. Gurdon, Khayam Javed, Munender Vodnala, Nigel Garrett

**Affiliations:** ^a^Wellcome Trust/Cancer Research UK Gurdon Institute, University of Cambridge, Cambridge CB2 1QN, United Kingdom;; ^b^Department of Medicine, Brigham & Women’s Hospital, Boston, MA 02115

**Keywords:** *Xenopus*, transcription factor, oocytes, dwell time, Ascl1 gene transcription

## Abstract

Some lineage-determining transcription factors are overwhelmingly important in directing embryonic cells to a particular differentiation pathway, such as *Ascl1* for nerve. They also have an exceptionally strong ability to force cells to change from an unrelated pathway to one preferred by their action. Transcription factors are believed to have a very short residence time of only a few seconds on their specific DNA or chromatin-binding sites. We have developed a procedure in which DNA containing one copy of the binding site for the neural-inducing factor *Ascl1* is injected directly into a *Xenopus* oocyte nucleus which has been preloaded with a limiting amount of the *Ascl1* transcription factor protein. This is followed by a further injection of DNA as a competitor, either in a plasmid or in chromosomal DNA, containing the same binding site but with a different reporter. Importantly, expression of the reporter provides a measure of the function of the transcription factor in addition to its residence time. The same long residence time and resistance to competition are seen with the estrogen receptor and its DNA response elements. We find that in this nondividing oocyte, the nerve-inducing factor *Ascl1* can remain bound to a specific chromatin site for hours or days and thereby help to stabilize gene expression. This stability of transcription factor binding to chromatin is a necessary part of its action because removal of this factor causes discontinuation of its effect on gene expression. Stable transcription factor binding may be a characteristic of nondividing cells.

## Significance

Some kinds of transcription factor proteins are very important in initiating and guiding cell fate differentiation. Overexpression of these factors can force many other kinds of cells to become muscle or nerve. Examples are MyoD for muscle and *Ascl1* for nerve. It is not known how long such a factor must remain bound to its binding site for it to have its function; this could be seconds, minutes, hours, or days. We have developed a procedure to determine the required residence time for the *Ascl1* nerve factor to have its function. This factor remains closely associated with its chromatin binding site for hours or days. This may be a general characteristic of such factors in nondividing (adult) cells.

Biochemical experiments have concluded that the dwell time of a transcription factor on its specific DNA site is remarkably short, on the order of seconds (1–5), and may involve oscillation of binding. It would be of considerable interest if the dwell time for some kinds of transcription factors were found to be enormously longer under certain circumstances. This is especially so if the duration of transcription factor binding to DNA is required for some kinds of normal cell lineage progressions and for the stability of cell differentiation. The results described here give evidence of a very long dwell time by the same molecule for a cell lineage–determining factor. This helps us to understand the mechanism of action of a transcription factor that guides cell fate and stabilizes cell differentiation.

We distinguish the dwell time of a factor at its site on DNA or chromatin from the site occupation time by a factor. The dwell time means the length of time for which the same molecule of a factor remains bound to its specific binding site. The site occupation time is the time for which a binding site is occupied by the same kind of transcription factor but not necessarily by the same actual molecule.

Some transcription factors are of great importance in directing embryo cells into their intended differentiation fate. The first example of this was MyoD, which has the ability, when overexpressed, to switch the fate of most kinds of cells into muscle (6). Subsequently, other examples of lineage-determining factors were and include *Ascl1*, a major neurogenic inducer in embryonic cells and one which causes cells to follow a neural differentiation pathway (7, 8). It can also make adult cells of different kinds become neural (9), like MyoD does for muscle (10). *Ascl1* is known to bind to a specific DNA sequence, CANNTG (11, 12), and is assumed, like the glucocorticoid receptor, to continually bind and dissociate from its DNA binding site, raising the question of its mechanism of action. We find here that the site occupation and dwell time of *Ascl1* can be much longer than has been generally believed. This may enable this factor to both initiate and stabilize a differentiated state and so give new understanding of the mode of action of a transcription factor in some kinds of cells.

## Results

### Experimental Design.

**Fig. 1.**
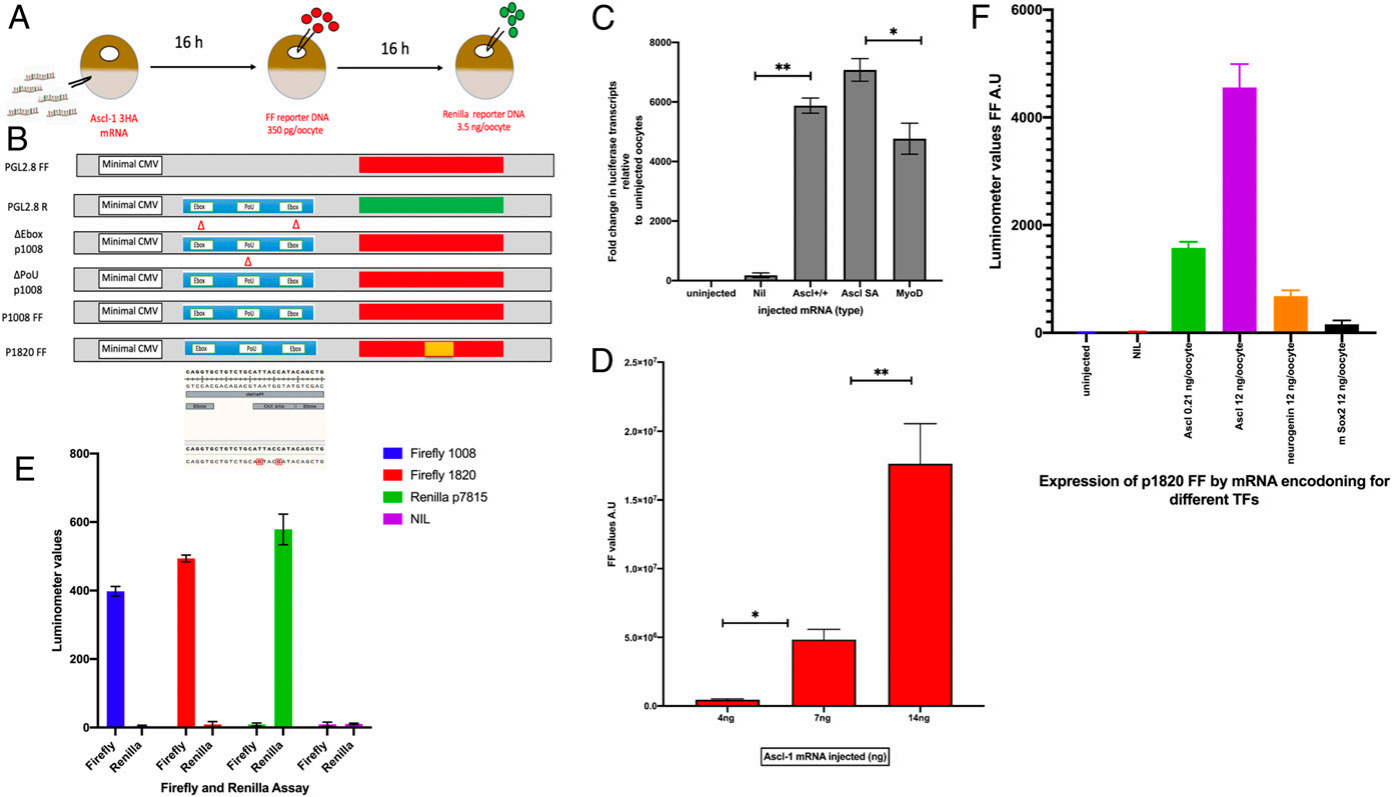
Experimental design. (*A*) The sequence of injections on living oocytes of *Xenopus*. The normal injection volume is 14 nL, aimed for the cytoplasm (mRNA) or the nucleus (GV). Nucleic acids were made up in water for injection. (*B*) DNA plasmid composition. The various plasmids used are derived from PGL4.28 (Promega). P1008 was provided by A. Philpott (Jeffrey Cheah Biomedical Centre Cambridge Biomedical Campus, Cambridge, UK) (ref. 25). The base pairs marked in red had been mutated as shown for the E-box and Pou sequences. (*C*) The mRNAs shown cause a substantial increase in the abundance of transcripts from the E-box–Pou–E-box domain of p1008 DNA. Ascl1 SA is a variant of mouse Ascl1 in which alanines have been mutated to serines. *P = 0.001; **P = 0.009. (*D*) Different amounts and kinds of Ascl1 mRNA induce a huge increase in Firefly expression of p1008 DNA. *P = 0.005; **P = 0.007. (*E*) The reporters FF for p1008 and p1820 respond strongly and independently to Ascl1 mRNA. The reporter Renilla responds similarly to DNA p7815 R. (*F*) Different kinds of mRNA induce specific reporter responses. Neurogenin (orange) has almost no effect and Sox2 (black) of mice and no effect at all on Firefly expression (red). Error bars represent the SEM in three independent experiments (*n* = 3).

This design needs explanation and validation. The residence time of a transcription factor determining cell fate is tested by induced gene response to a transcription factor and hence by function. The procedure for this use of oocytes is as follows (Fig. 1*A*). We inject messenger RNA (mRNA) encoding a transcription factor, in this case the neurogenic factor *Ascl1* (14), into the cytoplasm of a *Xenopus* oocyte, allowing the encoded protein to reach a desired concentration in the oocyte nucleus or germinal vesicle (GV). We then inject plasmid DNA, which has binding sites for the factor and an expression reporter, directly into the oocyte GV, so that it is immediately delivered to the nuclear environment of the *Ascl1* protein. We also use mRNA for a membrane-bound GFP, coinjected with the *Ascl1*-binding reporter DNA (p1008), to identify successfully injected oocytes (15, 16). The next day we assay each DNA-injected oocyte for expression of its reporter as an indication of the functional binding of the transcription factor to its specific DNA binding sequence. The next step is to inject plasmid DNA into the same already injected oocytes so that it can compete for any of the same factor that may have been released from the first plasmid DNA. If the residence time of the factor is short, the second DNA should compete for it and so cause expression of the second DNA. The extent to which the second DNA is expressed should therefore provide a measure of the residence time of the factor on the first DNA.

To what extent does this experimental procedure represent normal transcription in oocytes and in other cells? The injected DNAs are soon converted into nucleosomed chromatin, using the large store of oocyte histones (17–20) to which the factor binds, but are not integrated into the oocyte’s chromosomal DNA. Successful injection of template DNA into the oocyte nucleus is routinely accomplished in about 80% of attempts. We consider the injected nonintegrated plasmid DNA/chromatin to be the equivalent of the extrachromosomal nucleolar DNA always contained in a normal *Xenopu*s oocyte GV (21). A typical injection of plasmid DNA (p1008) at 100 pg/oocyte amounts to injecting 10^7^ molecules, and hence, this number of test genes is not much more than the 10^6^ endogenous extrachromosomal ribosomal DNA genes always present and transcriptionally very active in uninjected *Xenopus* oocytes. We know that both the E-box and Pou domains in p1008 DNAs are important for the transcription and translation of *Ascl1* and its reporter (22) (Fig. 1*B*). In typical trials, plasmid transcription is controlled by the *Ascl1* binding sites (E-box and Pou domains), so that assayable luciferase proteins are expressed. Plasmids lacking the E-box and Pou domains have a background expression of nearly 1,000-fold lower.

For many experiments we use the *Ascl1* binding reporter DNA plasmid p1008 FF, which causes a strong expression of a stable Firefly luciferase. Therefore, the luciferase activity that we record is the one which has accumulated from the time of DNA injection to the time when samples are frozen for analysis. In most cases this time is between 1 and 3 d. In other experiments we use the same plasmid with a very short half-life luciferase, namely, p1820 FF, but with exchanged reporter and regulatory sequences. Using cycloheximide to arrest protein synthesis, we find that the luciferase encoded by p1820 FF has a half-life of about 1.5 h as found by others (23) in oocytes, compared to about 50 h for an average protein (24). Using this DNA, we therefore see only the last 1 to 2 h of luciferase expression, even if the whole oocyte incubation period is up to 3 d. The half-life of luciferase mRNA synthesized in oocytes is about 5 h at 16 °C (α-amanitin assay). DNA p1820 and its reporter FF are not transcriptionally activated and expressed unless mRNA encoding *Ascl1* is present. The reporters FF and R (see Fig. 1*B*) are easily distinguishable and not overlapping.

A very important aspect of this oocyte assay is that the binding of *Ascl1* is assessed by its functional effect on gene expression. Most other assays for transcription factor association with a binding site measure binding visually or biochemically rather than by function.

### Characterization of the System.

The composition and expression of our p1008 and p1820 plasmid DNA are particularly important for the work described here. The DNA sequence to which the transcription factor *Ascl1* binds is an E-box–Pou-E–box motif, this CANNTG sequence being characteristic of several transcription factors, including *Ascl1*. The rest of the plasmid DNA (Fig. 1*B*) contains 12 CANNTG sequences (13, 25). Moreover, mutations in these two E-box parts of our p1008 plasmid reduce expression of the FF reporter down to about 4%, while the mutation of its Pou domain alone reduces its *Ascl1*-induced expression down to 80% (Fig. 1*B*). This means that this single DNA region is almost wholly responsible for the *Ascl1*-induced expression which we see. This is in contrast to many other transcription factor binding sites in DNA, where there are many such sequences in the genomic DNA, at least several of which may be important for expression induced in normal development (26). In p1008 and p7815, there are 12 CANNTG sequences, and we do not know whether one or many of these are normally required for the downstream effects of *Ascl1* to be seen.

**Fig. 2.**
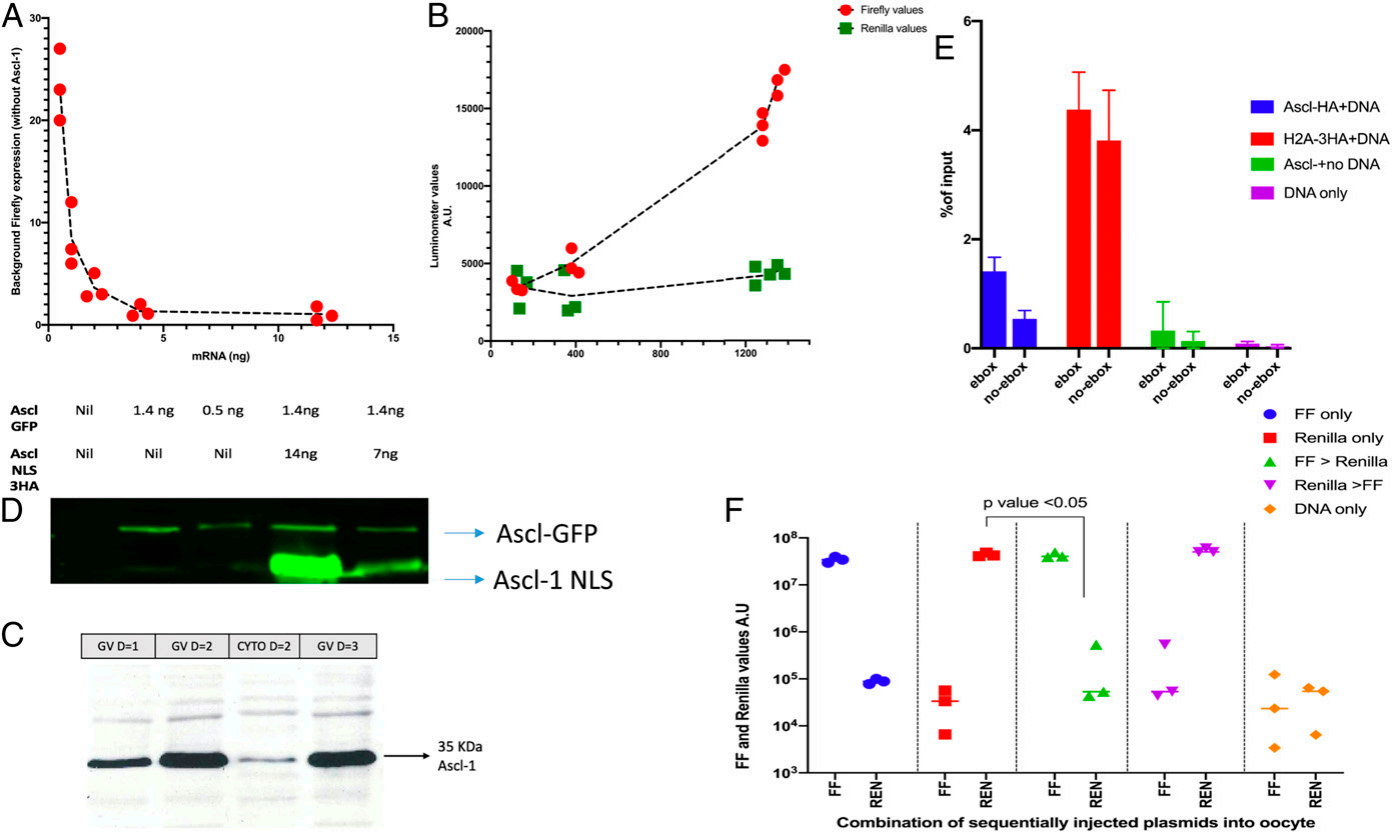
Characterization of gene expression induced by *Ascl1* mRNA in DNA-injected oocytes. (*A*) The background level of luciferase in oocytes that received DNA but no mRNA was 1 to 10% of the mRNA-induced level. (*B*) High levels of injected Ascl1 mRNA are sufficient to induce expression of a second injected DNA. (*C*) Ascl1 mRNA-encoded protein concentrates in the GV of injected oocytes and remains at a high level for 3 d. (*D*) A nuclear localization signal in Ascl1 mRNA helps to increase GV localization injected oocytes. (*E*) Injected DNA soon becomes associated with histone from injected histone mRNA. (*F*) The order of addition of DNA (FF or R first or sequential) gives the same conclusion: The first DNA dominates over the second. Error bars shows SEM for (*n* = 3) where applicable.

When we assay injected oocytes for transcripts from plasmid DNA p1008, we see an abundance of FF luciferase transcript within a day of DNA injection (Fig. 1*C*), and most of this RNA is dependent on a previous cytoplasmic injection of *Ascl1* mRNA, as well as being sensitive to α-amanitin. *Ascl1* and MyoD share a very similar binding sequence (22). In subsequent experiments we have chosen to assess our results by the amount of induced Firefly-tagged activity because this gives a measure of *Ascl1*-induced gene expression at the level of protein. The injection of mRNA encoding a transcription factor and plasmid DNA containing its binding region is followed by a very large increase in reporter luciferase activity of 10 to 100 times above the level in oocytes not injected with mRNA (Fig. 1*D*). Both FF and R reporter expression give a strong response, and there is no overlap between these two reporters (Fig. 1*E*). These reporters do not respond to injection of mRNAs encoding unrelated transcription factors, including neurogenin (Fig. 1*F*). In the absence of mRNA, there is a low level of FF luciferase expression from injected DNA, but this is usually only 1 to 10% of the level of luciferase seen after *Ascl1* mRNA when large amounts of mRNA are injected (Fig. 2*A*).

With large amounts of *Ascl1* mRNA both reporters, FF and R, are well expressed (Fig. 2*B*). Enough *Ascl1* protein is generated to allow transcription of sequentially injected reporter plasmids. The induced *Ascl1* protein concentrates in the GVs of recipient oocytes when it settles to a steady concentration for a few days (Fig. 2*C*). The localization of *Ascl1* protein in the GV is enhanced by the use of a nuclear localizations sequence (NLS) signal in the *Ascl1* mRNA (Fig. 2*D*). To confirm previous work, we have checked that injected DNA becomes quickly associated with histones (Fig. 2*E*). The order of injection of the two DNAs (FF or R) does not affect the strong response of the DNA to mRNA injection (Fig. 2*F*). In the absence of mRNA injection there is a low level of FF luciferase from the injected DNA, but this is usually only 1 to 10% of the level of luciferase seen after *Ascl1* mRNA when large amounts of mRNA are injected (Fig. 2*A*). After mRNA injection into oocytes, *Ascl1* protein soon accumulates in oocytes, where it settles to a steady concentration for a few days. Western analysis of isolated GVs has also enabled us to show a steady concentration of *Ascl1* protein in the GV of injected oocytes over 3 d and that the concentration of *Ascl1* protein in the GV is 10 to 20 times above that in the cytoplasm (Fig. 2*C*).

To determine the duration of DNA site occupation by a transcription factor, we subsequently inject a second plasmid DNA with the same transcription factor binding site to act as a competitor, but with a different reporter. This is p7815 with a *Renilla* reporter (Fig. 1*B*). If the *Ascl1* factor is not stably bound to the first plasmid DNA, it will be released and then bound by the second (competitor) DNA, especially if a competitor DNA is in a high concentration and if the concentration of *Ascl1* mRNA is limiting. We need to be sure that both the test reporter DNA plasmid and the competitor plasmid respond similarly to overexpression of the mRNA that encodes *Ascl1*. To further characterize this experimental system, we also want to be sure that oocytes that have already received a cytoplasmic injection of mRNA and a GV injection of DNA can, nevertheless, transcribe and express a second GV injection of another DNA a day later. We initially tested this with a first injection of competitor DNA, followed by reporter DNA, to see if, as expected, the reporter DNA expression is not precluded by the first injected DNA. In these experiments, we used a large amount (2 ng or more per oocyte) of *Ascl1*-encoding mRNA. We see that expression of the second DNA (p1008 or p1820) increases substantially as a high level of mRNA is supplied (Fig. 2*B*) to give these conditions of excess mRNA. This excludes the possibility that under these conditions, a second DNA is either degraded or converted into a nonfunctional form.

The conclusions from many experiments, which gave very similar results, are as follows: 1) Two sequential injections of DNA into the same GV can result in meaningful transcription of two plasmid DNAs, as long as there is a large amount of *Ascl1* protein from injected mRNA so that the two DNAs are not in competition. 2) The second DNA injection in these experiments normally includes a GFP membrane-associated plasmid DNA which shows that the success of GV injection is generally over 80% and enables injections that miss the GV to be excluded. 3) The overall conclusion from these experiments is that oocyte injection provides a valid assay for a functional transcription factor effect on its DNA binding site and on the consequential gene expression. It is important to appreciate that the amount of mRNA in these initial experiments was large enough for this not to be limiting (Fig. 2*B*). Therefore, even when all *Ascl1* binding sites on the first DNA are occupied by *Ascl1*, there will still be enough of the *Ascl1* factor in the test oocytes to permit some transcription and expression of the second injected reporter DNA.

### Competition Experiments.

#### Limiting mRNA.

**Fig. 3.**
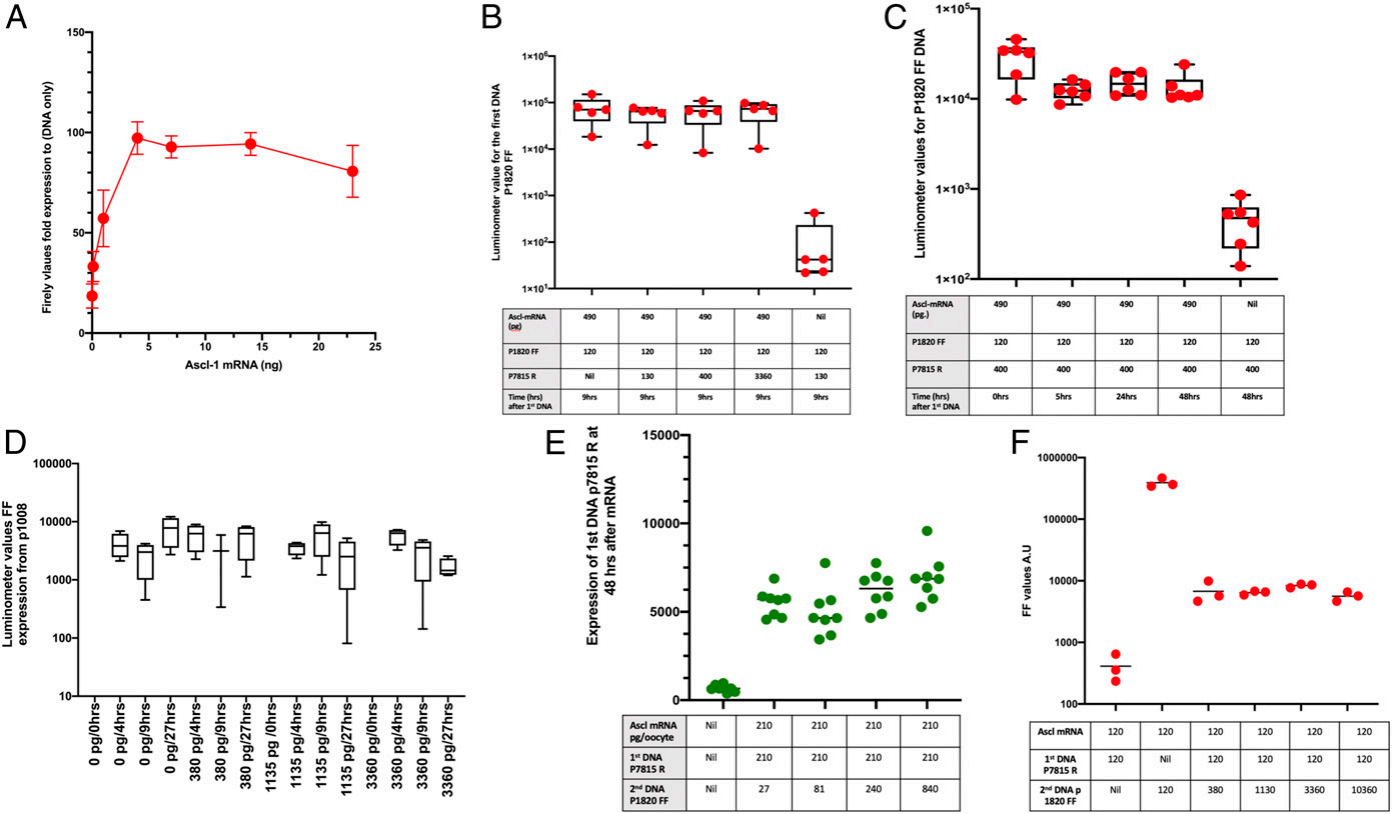
Lack of competition for sequentially introduced DNA binding sites. (*A*) Fifty picograms of plasmid DNA are nearly saturated by 4 ng of Ascl1 mRNA. In most samples of oocytes, 500 pg to 1 ng of Ascl1 mRNA per oocyte containing 210 pg of p1820 DNA was sufficient to ensure that the synthesized protein limits the amount of luciferase reporter seen. Beyond this level, no further reporter expression is obtained by injecting a further amount of DNA or RNA. (*B*) Increasing the amount of a second competitor DNA up to 3,360 pg/oocyte does not increase the amount of the first DNA’s reporter (p1820 FF). (*C*) A lack of competition is seen by a second DNA if the duration of incubation is prolonged for 2 d after the first injection. (*D*) Summary of results from 17 frogs in different experiments, showing that no significant reporter expression is obtained from a second injection of DNA. The synthesized DNA was injected at 0.2 to 12 ng/oocyte. (*E*) After Ascl1 mRNA at 210 pg/oocyte, we inject competitor DNA 7815R, also at 210 pg/oocyte. The next day, expression p1820 FF is injected at concentrations from nil to 840 pg/oocyte. (*F*) The amount of expression DNA p 1820 FF is the same in all samples, but the amount of p7815R as first DNA, as a competitor, shows the expected increase.

We now need to make the amount of *Ascl1* protein limiting to create competition between the two DNAs for the *Ascl1* factor derived from injected mRNA. At low amounts of injected *Ascl1* mRNA, reporter Firefly luciferase activity increases in proportion to the amount of mRNA injected (Fig. 3*A*), and a further increase in the amount of *Ascl1* mRNA makes very little difference to the induced FF expression. In most batches of oocytes, 1 ng of *Ascl1* mRNA or less behaves as a limiting amount and saturates subsequently injected DNA. Increasing amounts of injected DNA give a small, but not proportionate, increase in the amount of induced luciferase reporter expression. The amount of mRNA needed for a limiting level varies between batches of oocytes but is generally <4 ng/oocyte (Fig. 3*A*).

#### Expression DNA first.

Under the conditions of 490 pg mRNA/oocyte, we injected p1820 FF as the first DNA at 120 pg/oocyte and then, 1 d later, injected a second DNA, p7815 R, at increasing concentrations (Fig. 3*B*). In further tests, we increased the duration of incubation after the second DNA to up to 48 h from the time of the first DNA (Fig. 3*C*). In Fig. 3*D*, we summarize the results of 17 independent experiments. Note that the scale in Fig. 3 *B*–*D* is logarithmic to draw attention to the magnitude of the difference between control and experimental samples. In all cases, we see an extraordinary stability of *Ascl1* binding to its DNA or chromatin region and no evidence of its dissociation of *Ascl1* from DNA or chromatin within 2 d and under conditions of very large competition.

#### Confirmation by inverted order of injected DNAs.

To verify the overwhelmingly dominant effect of the first DNA over a subsequent DNA, we inverted the order of addition of DNAs (Fig. 3*E*). We injected a first DNA (tagged with *Renilla*) followed by increasing amounts of a second DNA FF. We saw no change in expression of the first DNA. This is true even with a huge excess of the second DNA concentration (10,360 pg/oocyte) and an incubation time of 24 h after the supply of competing DNA (Fig. 3*F*). Therefore, again, the second DNA (p1008 FF) in excess is not able to increase its own expression or to change expression of the first DNA (p7815 R). This means that the *Ascl1* transcription factor did not dissociate to any extent from the p1008 DNA since any that had been released would have been taken up by the first DNA and increased its expression.

**Fig. 4.**
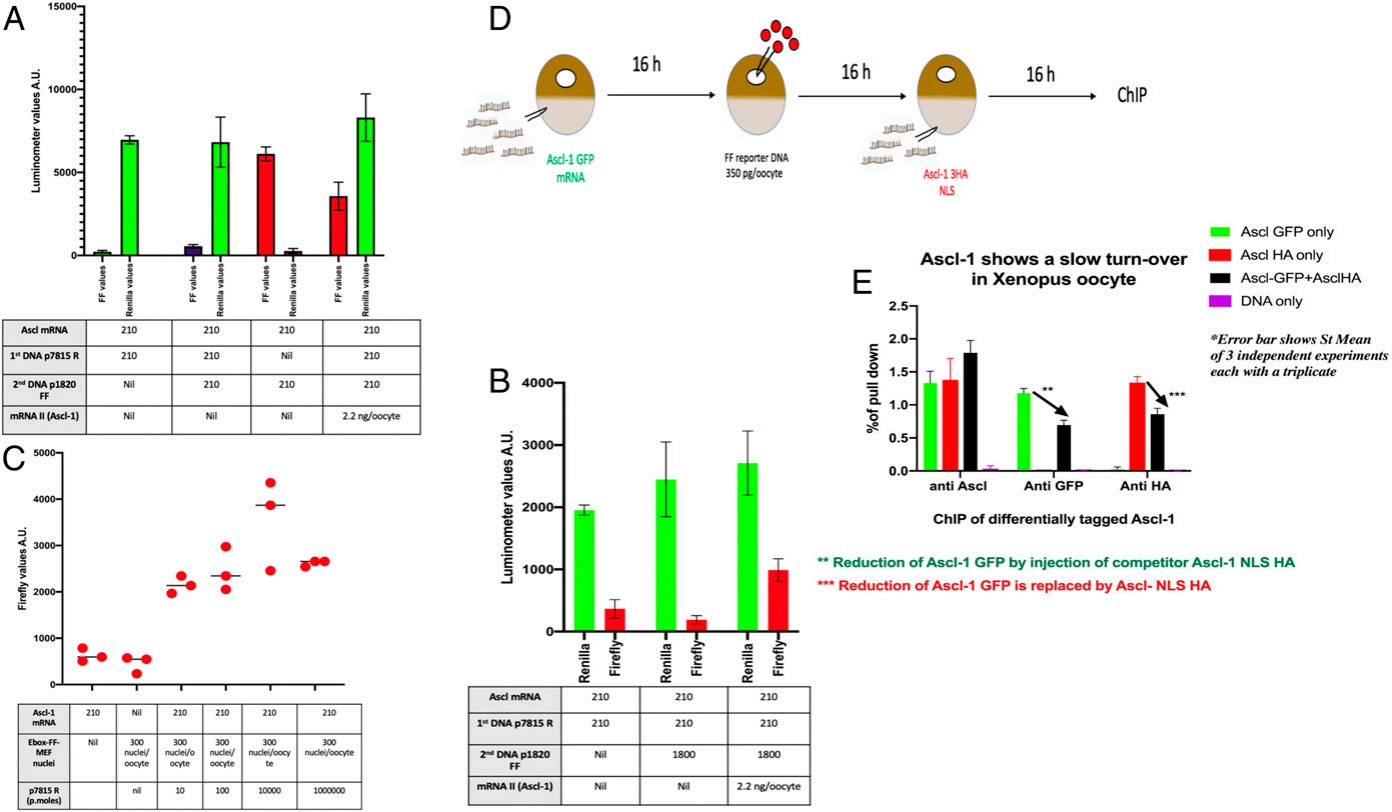
Rescue of nonexpressing second DNA. (*A*) It starts with the usual injection of Ascl1 mRNA at 210 pg/oocyte. After 8 h, a GV injection of the first DNA (p7815R) is made. Twelve hours later, another GV injection of DNA is made, this time of p1820 FF at 210 pg/oocyte, as the second DNA. As expected, this second DNA is poorly expressed because of the earlier injection of the first DNA. However, if a second injection of Ascl1 is now made with 2.2 ng/oocyte to raise the level of Ascl1 protein above the limiting amount, it now causes a strong expression of the second DNA. This increased FF values from 1,346 to 6,900. This compares to the high initial level (13,427) of expression of p1820 FF DNA if the reexpression of the previously expressed second DNA (p1820 FF) has now been raised by five times had been no initial expression of it by 7815R. (*B*) Confirmation of second DNA rescue due to a further supply of Ascl1 mRNA. The experimental design is similar to that in A, except that a greatly increased amount of p1820 FF was introduced. Even now there is no indication of release of Ascl1 from the first DNA, to which it seems to be stably bound. The second injection of mRNA II causes a large increase in expression of the second (repressed) DNA. After Ascl1 mRNA and subsequent competitor p7815R DNA, both at 210 pg/oocyte, p1820 FF was injected 20 h later at the increasing picogram amounts shown. FF was analyzed 24 or 26 h later (the two time point values have been combined). The increasing amount of the second (p1820) DNA does not reduce the response of the initial competitor DNA expression (red). (*C*) Transplanted nuclei with an integrated E-box–Pou–E-box DNA sequence. One day after nuclei injection, p7815 Renilla competitor DNA was injected into the GV in different amounts. Thirty-six hours after that luciferase, FF or R, was scored. The figure combined with the experimental design shows the luciferase values for FF were about the same independent of additional competitor DNA ranging from 0 to 106 molecules per oocyte GV (Fig. 5*A*). (*D*) Protein competition design; two differentially tagged Ascl1 proteins compete for the same binding site. (*E*) Using two kinds of proteins together with ChIP analysis shows occupation of the Ascl1 binding site in DNA by each kind of protein competitor. Using p1008 FF as a competitor, we see a reduced binding of it to DNA down to about 70% of the high competitor level. If we use Xklf2 mRNA to compete with the Ascl1 mRNA, we see a reduction of binding from 100 to 80%. Therefore, the binding of Xklf2 competes almost as well as Ascl1 for the binding site on DNA. The competitive binding of Xklf2 is therefore not competing directly with Ascl1 mRNA.

We emphasize that in the design of these experiments, we do not require expression of the second competitor DNA; it has only to be able to bind any available *Ascl1* protein, including that which might be released by a short dwell time on the first injected DNA. Nevertheless, we want to be sure that a second injected DNA is in a functional state. It could be imagined that when it does not compete with a first DNA, a second DNA could be converted, directly or indirectly, to a noncompetitive state. We have tried therefore to rescue expression of the noncompeting (second) DNA as follows (see Fig. 4*A*).

#### Rescued expression of the second DNA.

We prepared oocytes with limiting *Ascl1* mRNA to 210 pg/oocyte. This was followed by injection of a first DNA, *Renilla* (p7815 R), which was expressed. This was then followed by a second DNA injection of p1820 FF. As found before, this second DNA was poorly expressed because of the preceding (p7815 R) DNA, in this example, FF values of 1,346 compared to 6,900 for no first DNA (Fig. 4*A*, second column). However, when we supplied a second injection of *Ascl1* mRNA (2.2 ng/oocyte) at the same time as the second DNA so that *Ascl-1* protein was no longer limiting, this increased expression of the second (repressed) DNA p1820 FF from 1,346 to 13,427, an increase of 10 times (Fig. 4*A*, second column versus fourth column). An independent repeat of this experiment with different amounts of injection gave a similar result (Fig. 4*B*). Therefore, we have been able to enforce expression of a second DNA that is repressed by competition, using an increased supply of *Ascl1* mRNA. Two further such experiments gave a similar result. Thus, we find that when a second DNA is not well expressed, following an injection of the first DNA, it is able, with high doses of extra *Ascl1* mRNA, to be bound by expressed *Ascl1* protein.

The rescuing effect of the second *Ascl1* mRNA injection is not complete. It reaches about half the maximum level that can be reached by *Ascl1*-induced FF expression if there had been no competitor DNA at all. If we try an RNA rescue experiment using a complete GV extract including all of the numerous transcription factors present in a normal oocyte GV, this can also have some rescuing effect.

### DNA Response Element Integrated into Chromosomal DNA.

We now ask whether the stable occupation of a binding site on DNA or chromatin by a transcription factor as observed in our extrachromosomal plasmid DNA experiments above is also true of the same DNA binding sequence integrated into chromosomal DNA. The experimental design is similar to that used in our previous plasmid experiments (Fig. 3*B*) where DNA was transfected. We transfected the whole E-box–Pou–E-box response element from plasmid DNA p1008 FF into chromosomal DNA of a mouse embryonic fibroblast (MEF) cell line commonly used in our other oocyte experiments. We transplanted these MEF nuclei carrying a transfected E-box–Pou–E-box DNA for *Ascl1* binding into oocytes already expressing the *Ascl1* transcription factor after mRNA injection, as in our plasmid experiments. The enhancement of p1008 FF expression in transplanted nuclei shows the same 25-fold increase over non-mRNA-injected samples, as is seen in equivalent plasmid DNA experiments. We then carried out competition experiments as described above for plasmid DNA. The same conclusions are reached. The competitor plasmid DNA p7815 causes no detectable reduction in Firefly luciferase encoded by the DNA-transfected injected nuclei (Fig. 4*C*). Remarkably this is even true if the amount of competing DNA is increased from 10^2^ to 10^6^ (Fig. 4*C*). This result was seen at both 24 and 48 h after injection of competitor DNA (Fig. 4*C*). We conclude that a chromosomally integrated *Ascl1* binding site behaves the same as in our extrachromosomal plasmid DNA experiments; in each case the duration of the transcription factor binding site occupation is remarkably long and resistant to a large excess of competitor DNA. Again, there is no evidence for the release of bound *Ascl1* protein from DNA or chromatin DNA, even after many hours.

### Protein Competition.

Since we have reached an unexpected conclusion different from that of previous work, using an unconventional assay, we have sought to test our conclusion by another procedure (Fig. 4*D*). This involves a protein competition experiment. We cannot use the same DNA-encoded FF reporter assay as above because a competing *Ascl1* protein would also induce expression of the same DNA Firefly reporter and so obscure any competition effect that might exist. We have therefore used chromatin immunoprecipitation (ChIP) analysis to determine the binding of *Ascl1* protein to its DNA binding site. We start with an injection of *Ascl1*-GFP mRNA, which is soon translated into *Ascl1* protein. After nuclear injection of DNA p7815R into the GV, we then inject mRNA for an *Ascl1* tagged with HA (Fig. 4*D*) to compete for binding with the first injected *Ascl1*-GFP mRNA and ask whether, by ChIP, an excess of *Ascl1*-HA can displace the other already bound *Ascl1*-GFP. This would happen only if the first bound *Ascl1*-GFP protein were to have a short dwell time on its DNA. We see a reduction in *Ascl1*-GFP binding compared to no competition at all, but only by 30%, and this is with a 10-fold excess of competing RNA and for a 23 h incubation period (Fig. 4*E*). However, this could be due to an unspecific effect of a large amount (10 ng) of competing RNA. To test this, we have competed the first *Ascl1*-GFP with an mRNA encoding the completely unrelated xklf2-HA, which has shown strong protein expression in our other unrelated experiments. We now see a reduction in *Ascl1*-GFP binding, but only by 20% (Fig. 4*E*), and this is only 10% different from competition with the specific competitor *Ascl1-*HA. Therefore, the main effect of this DNA binding experiment results from a high amount of mRNA. We have checked that the amount of competitor *Ascl1*-HA protein in this experiment was as high as expected from its high mRNA injection.

We point out that even if there is any real difference between the DNA and protein competition results, the main conclusion is the same. It is that a dwell time of *Ascl1* protein in these living cell experiments is very long indeed compared to previous results of others with the glucocorticoid receptor.

### A Long Residence Time Is Also Seen with the Estrogen Receptor.

We pointed out in the introduction above that previous work in this field has been done with receptors which govern gene expression and that need to change frequently according to conditions; these include the estrogen receptor (3, 27, 28). We now ask whether the long residence time reported for *Ascl1* in oocytes is also true of the estrogen receptor when tested in oocytes by a competition assay.

**Fig. 5.**
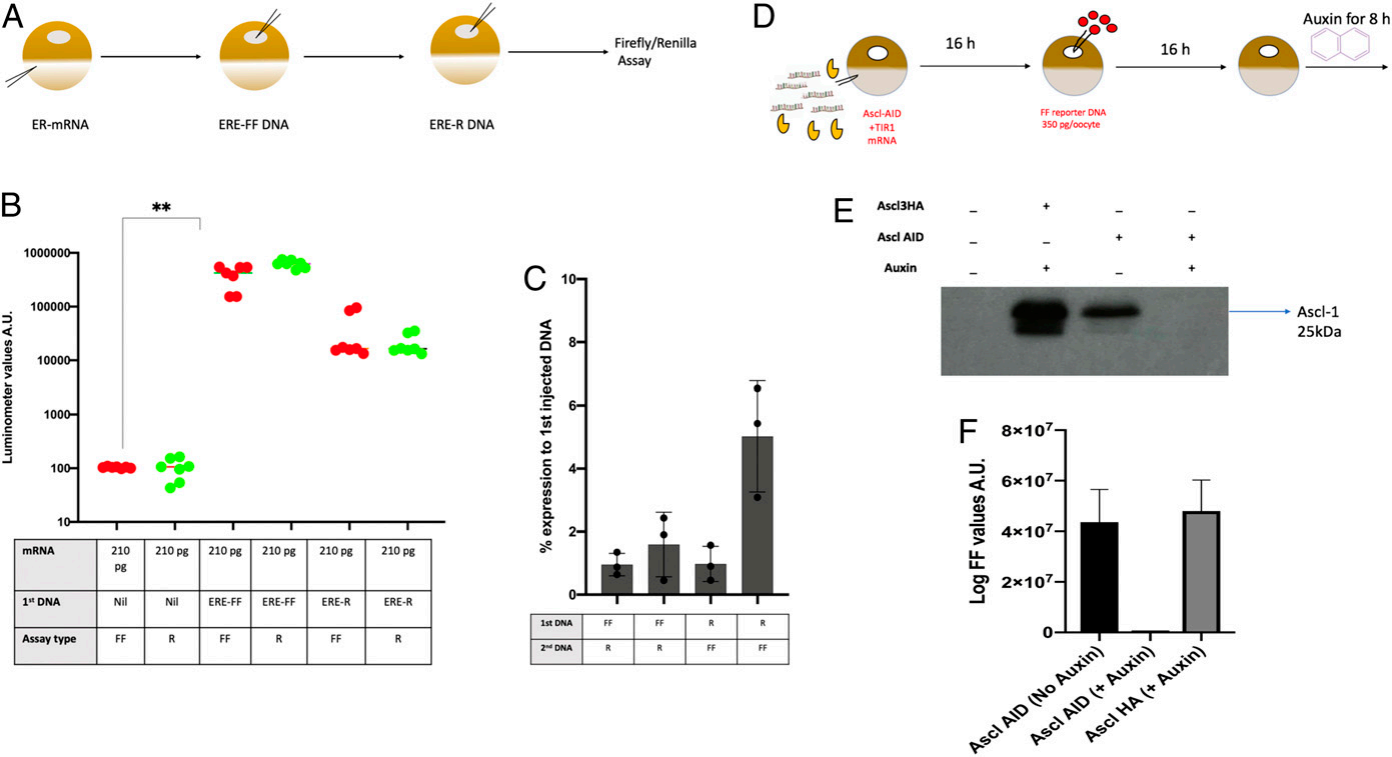
An estrogen receptor induces a very strong and stable expression of estrogen reporter gene expression. (*A*) Design of experiment. Estrogen response mRNA was injected at 210 pg/oocyte. One day later, estrogen response element DNA at 210 pg/oocyte, tagged with either FF or R, was injected into the GV. One day after that single oocytes were assayed for FF or R fluorescence. (*B*) Estrogen mRNA gives a very strong response if followed by estrogen response element DNA. Thus, estrogen response element tagged with FF increased by 106-fold (red), and that tagged with R increased by about 100-fold (green). The first and second columns show incubations for 48 h between mRNA injection and assay; for the fifth and sixth columns, the incubation period was 72 h. (*C*) An estrogen receptor bound to its estrogen reporter DNA resists competition by DNA was a differently tagged reporter. The percentage values given show that the second DNA (whether tagged by FF or R) gives only a 5% expression compared to the same DNA injected first. The same effect as for Ascl1 is seen: Whichever DNA is injected first dominates over the second DNA. (*D*) A functional necessity for persistent Ascl1 protein on E-box chromatin: auxin depletion of Ascl1 protein. The design of the experiment is as follows: Oocytes were injected with 1.0 ng Ascl1-3HA mRNA. The next day, 210 pg of p1008 DNA were injected, and 1 mM auxin was added to the medium. (As a negative control, Ascl1-HA mRNA [no AID] was included.) Oocytes were processed for Firefly analysis. The results show that both Ascl1-HA and Ascl1 AID were strongly expressed 72 h after the first mRNA. (*E*) Western analysis to show auxin depletion of protein. Injected oocytes were separated in oil into GVs and cytoplasms; groups of 10 oocytes were then frozen and analyzed for Western blot procedure. (*F*) Complete removal of protein by auxin is seen. Injected oocytes were analyzed as in previous samples for FF values.

We used mRNA for an estrogen receptor ER1 and estrogen response elements tagged with Firefly or *Renilla* (Fig. 5*A*). The first DNA was injected 20 h after the mRNA; the second (competitor) DNA was supplied in excess 1 d after the first DNA, and samples were collected 24 to 48 h after the second DNA. We also find that in an experimental design similar to that carried out for *Ascl1*, the provision of an estrogen reporter element elicits an enormous enhancement of estrogen reporter element expression (Fig. 5*B*). Estrogen does not need to be added. We find that a second DNA is minimally expressed if it is induced a day after the equivalent, but differently tagged, first DNA (Fig. 5*C*). This result is seen whether the Firefly- or *Renilla*-tagged DNA is injected first (Fig. 5*C*). We find that a large amount of competitor DNA does not reduce expression of the first DNA even though it could do so without its own expression. Thus, the reporter expression of the first DNA is not reduced by the coinjection or not of a second DNA (Fig. 5*C*).

We conclude that the long residence time and long duration of resistance to DNA competition for an estrogen receptor are similar to those for the *Ascl1* receptor. There is therefore some special characteristic of an oocyte or its components which stabilizes the binding of a receptor to give a very long duration of induced gene expression.

### The Continuing Presence of *Ascl1* Is Required for Its Induced Stable Gene Expression.

Following the conclusion that *Ascl1* has an unexpectedly long dwell time on its chromatin, the next key question is whether this is of functional importance. We need to ask if the continuing presence of *Ascl1* is required for its long-term stable effect on induced gene expression. We consider the possibility that *Ascl1* is no longer present and that its stable effect on chromatin is taken over by another mechanism. The most direct test of this question is to remove *Ascl1* after its stabilizing effect has been established and to assess the binding of *Ascl1* protein by ChIP analysis to check that it has been removed. If so, does this reduce the suppression of a second DNA and hence show that the long dwell time that we find is part of the function of the transcription factor? We have been able to do this by making use of the auxin-dependent degradation procedure of Tan et al. (29) and Natsume et al. (30). Oocytes were prepared by the injection of *Ascl1*-HA-AID mRNA, followed the next day by p1008 FF DNA (Fig. 5*D*). On the following day, auxin was added to the oocyte medium. Soon after that, competition plasmid DNA (7815R) was injected into the GV, and a few hours later samples were collected for ChIP analysis. The Western results, in Fig. 5*E*, show that the *Ascl1* protein was eliminated after auxin treatment. In the same samples, Firefly expression was also reduced to a negligible level compared to the nil-effect no-auxin controls and was not reduced at all when a nondegradable *Ascl1* was used (Fig. 5*F*). We therefore see that the induced expression of DNA + FF is dependent on the continuing presence of *Ascl1* and that its stabilizing effect, resistant to competition, is not replaced by another mechanism.

We conclude that the presence of the *Acl1* transcription factor is required for its long-term and stable gene-inducing effect. This provides a functional test for the long-term and site occupation stability of the *Ascl1* transcription factor bound to chromatin.

## Discussion

The bottom line of this work is that the early bird catches the worm (31). We find that in a nondividing cell, two transcription factors that bind to their specific DNA sites can remain for several hours or days and that this largely resists competition from similarly specific DNA sequence (or chromatin). This gives insight into the mode of action of a transcription factor. This result is very different from the common view that transcription factors have very short residence times of seconds or minutes.

For a number of years, Hager and colleagues have used many different methods to estimate the time the glucocorticoid receptor remains bound to its DNA binding sequence. The dwell times they have found are surprisingly short, namely, from less than 1 s to a few minutes (2, 27, 28, 32–37). Work with the estrogen receptor has given similarly short times. In accord with this finding is the oscillatory behavior of Ascl1 expression in proliferating neural progenitor cells (38). Most recently, very short dwell times were found for factors that regulate gene expression in early rapidly dividing *Drosophila* embryos. In these cases genes need to be able to respond rapidly to changing metabolic conditions as in embryos (37). Using a gel shift dissociation assay, Fong et al. (12) found a binding time of 2 to 3 min for MyoD (CAGGTG) and NeuroD (CAGATG) in cultured mammalian cells. One of the longest dwell times described is that of the binding to the heat shock gene in differentiated *Drosophila* cells, where a replacement time of 1 h was seen (39). Individual transcription factor components can also exchange very rapidly, such as TFIID (40). The results reported here are clearly not in accord with this previous work, which was mostly done with dividing and proliferating cells. The dwell time in our experiment is hours, or even days, and is seen even when a huge excess of a competitor is directly introduced into a nucleus.

The idea of a long residence time for a transcription factor and resistance to competition was already discussed many years ago in the widely appreciated work of Brown and colleagues (41–43) using an entirely different system from what we describe here. The system used by Brown and colleagues was an in vitro transcription of 5S ribosomal genes by RNA polymerase III. In vitro transcription is successful using RNA pol III, but no in vitro reinitiating transcription by pol II has been described, though it does take place in living, injected *Xenopus* oocytes, which also give accurate transcription with RNA pol III (44). In *Xenopus* oocytes, there are 20,000 genes that encode the oocyte-specific 5S ribosomal gene, and these are actively transcribed in oocytes but not at all in somatic cells, whose 5S ribosomal gene transcription is undertaken by 600 somatic genes (45). It was suggested that the nontranscription of the oocyte 5S genes could be explained by a repression effect of histone with a differential binding affinity of the transcription factor TF IIIA for oocyte versus somatic genes (43).

How can we account for this difference between our results and those described before? DNA injection in *Xenopus* oocytes was used, long ago, to find some competition between two different kinds of DNA in induced gene expression (25, 46, 47). But this work does not relate to the mode of action of identified transcription factors. The most obvious difference is in the assays made. Previous work concentrated on transcription factor binding, whereas our results measure expression induced by the transcription factor. Expression seems to us most relevant for cell lineage and cell fate decisions. This difference does not, of course, explain the very short dwell times seen in previous work.

**Fig. 6.**
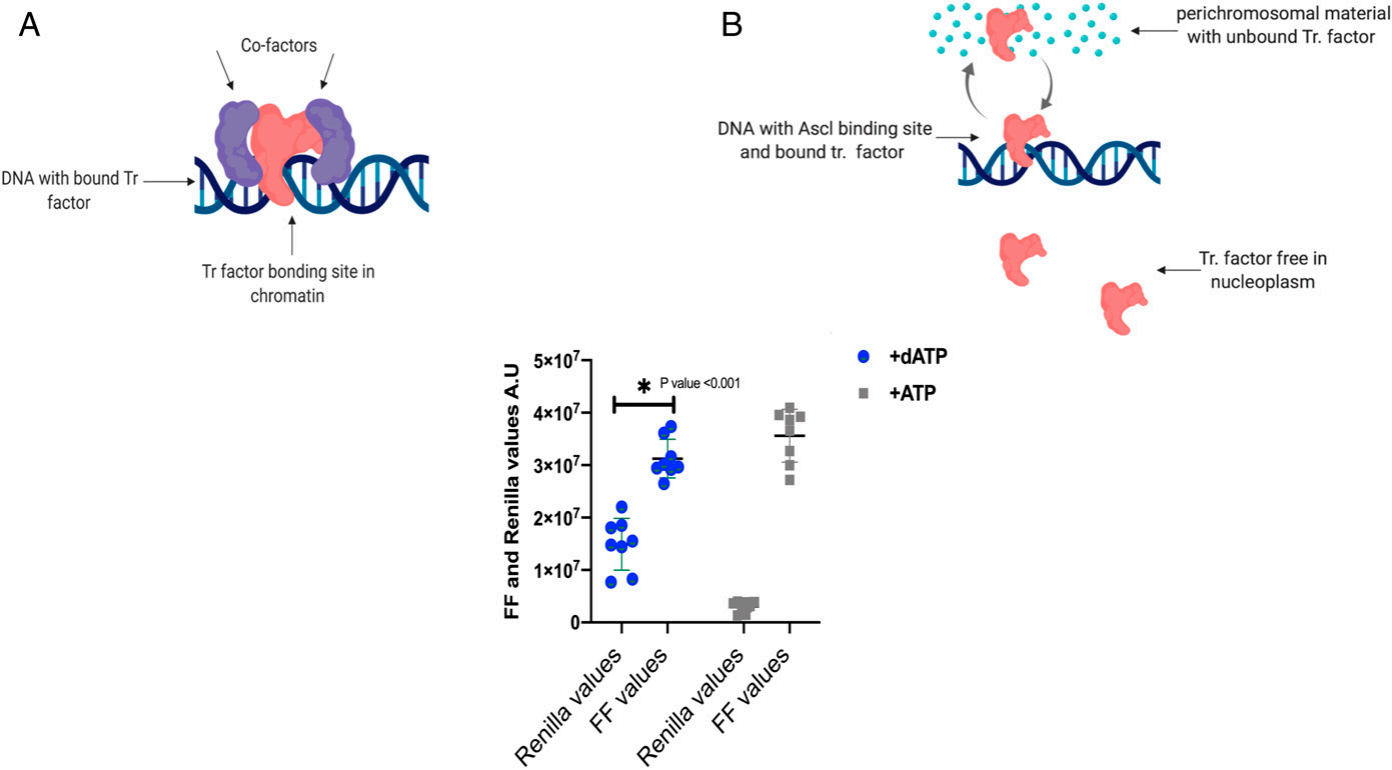
Hypothesis based on cofactors for stabilization. (*A*) Cofactors are assumed to bind close to the bound Ascl1 protein and to stabilize Ascl1 binding for several hours. (*B*) Hypothesis to show the phase separation concept. Bound Ascl1 protein is supposed to dissociate from DNA and to rapidly move between its DNA binding site and nearby liquid phase–separated material. (*C*) Effect of ATP overexpression. mRNA and subsequent DNA plasmid (p1820) were injected as usual. Then a second DNA (p7815R) and ATP at 25 μg/mL (15 nL) were injected. The next day FF expression was assayed as done previously. We see that the provision of ATP induces strong expression of the otherwise unexpressed second DNA.

The most likely explanation is by cofactors which could bind close to *Ascl1* (Fig. 6*A*). However, another possible explanation for this (unexpectedly) long dwell time and resistance to competition in our DNA injection experiments would be for the first injected DNA to become associated with another nearby nonchromosomal complex, where it could take up the limited supply of *Ascl1* protein, so that there would not be enough of this protein to give transcription of a second injection of DNA (Fig. 6*B*). For example, transcription complexes in amphibian oocytes are believed to be in liquid phase–separated complexes (48, 49). Support for the idea that an *Ascl1* chromatin could be subject to phase separation comes from our finding that the injection of adenosine triphosphate (ATP) with the second DNA has the strong effect of making it well expressed in a competition test (Fig. 6*C*). Hayes et al. (49) pointed out that ATP has both hydrophilic and hydrophobic properties and could therefore interfere with phase separation. Future work will explore the likely role of a cofactor, phase separation, or both in stabilizing *Ascl1* in chromatin.

A very interesting possibility is that there are two modes of transcription factor binding, namely, one for short-term binding in dividing and proliferating cells, including oscillation (5, 38, 50), and the other for stable gene expression in nondividing (including oocytes and adult) cells. Stable binding could help to secure long-term gene expression and the differentiated state of cells. It is an attractive aim to try to identify any cofactors that can stabilize transcription factor binding. An important early paper (51) discussed some general transcription factor principles.

## Materials and Methods

### *Xenopus* oocytes.

Xenopus oocytes experiments were approved by the University Biomedical Services at the University of Cambridge and compiled with UK Home Office guidelines (Animal Act 1986). The frogs used to supply oocytes were reared from fertilized eggs in our laboratory. The oocytes are taken from the ovary under terminal anesthesia. After subcutaneous (s.c.) injections of MS222 (methane sulfonate) frogs are kept on ice while fully anesthetized, and the ovary is then removed. The frogs do not recover from the terminal anesthesia.

Defolliculation of oocytes is required before they can be injected. This is done by incubating small groups of oocytes in Liberase (research grade supplied by Roche) for 2 h at room temperature. The individual oocytes retain one inner layer of follicle cells but no blood vessels or blood cells. They are then incubated in modified Barth saline solution (52) overnight with 0.1% bovine serum albumin (BSA) in the medium. The BSA helps to reduce the potentially damaging effect of the Liberase solution on the oocytes. The few dead oocytes are removed before injection. Before cytoplasmic injections, material is deposited equatorially. For germinal vesicle injections, a needle is introduced at right angles to the surface of the animal pole. The success of injection material into the germinal vesicle of an oocyte is about 80%. To monitor the success, we inject a membrane GFP encoding plasmid DNA together with the rest of the sample into an oocyte GV (2, 37) Oocytes successfully injected into the GV are seen as bright green fluorescence on the whole oocyte the day after injection. For GV injection, see below in *Oocyte GV Injection* (7).

### Equipment.

Glass needles are pulled out by machine, and the tips are given a sharp point and smooth opening using a microforge constructed in our laboratory (53). The opening of an injection needle is about 5 μm in diameter.

### Culture Medium.

We use Modified Barth's Solution (MBS) saline solution (52) for this supplemented with 0.1% BSA. Injected oocytes are cultured at 16 to 18 °C.

### DNA Constructs.

We inject plasmid DNA at the stated concentrations. The various constructs we use are based on PGL 4.28 (Promega). For most purposes we use p1008 with a C-terminal Firefly reporter. In some cases the Firefly reporter is replaced by a *Renilla* reporter. Plasmid p1008-SA has seven serines in the parental DNA replaced by alanines. The DNA for injection is in a double-stranded circular form. Constructs for the estrogen work were donated by J. Carroll (University of Cambridge, Cambridge, UK), from whom further information can be obtained (Jason.carroll@cruk.cam.ac.uk). The cell line with an E-box–Pou–E-box integration appears to have only one integration site as a result of selection during the preparation of this cell line.

### Nucleic Acid and Protein Extraction.

Nucleic acid and protein extraction is done by our standard procedures, as described by Halley-Stott et al. (15).

### Statistics.

In most of this work the effects we see are very large; for example, we see a 100 times larger effect over background than when mRNA or DNA is omitted (so we do not see the need for statistical analysis); see, for example, Fig. 2*F*. The statistical tests for Fig. 2 *E* and *F* were Student’s *t* test and two-way ANOVA.

### Oocyte GV Injection.

Since the position of the GV in recipient oocytes is not visible, some practice is required to obtain an 80% or better rate of success in depositing material in the GV of living oocytes. In some experiments, we coinject a plasmid DNA with a GFP tag such that the oocyte’s membrane is visibly green under fluorescence, and the oocytes not showing this GFP are excluded from subsequent analysis. The chance of a second DNA being deposited in the same part of the GV as the first DNA is not always achieved, and the gel-like consistency of the GV means that the two DNAs do not always move to the same part of the GV. The variability of luminometer values for the two FF or R fluorescences depends on how effectively the second GV injection was deposited in the same part of the GV as the first injection. Moreover, the values for oocytes with or without a second DNA injection are sufficiently large that the conclusions are not affected by this uncertainty (see Fig. 6).

### Data Availability.

All the data used in this manuscript has been mentioned in the text. The data that support the findings of this study are available from J.B.G. upon reasonable request.
